# Supplementary material for: Multi‐dataset identification of innovative feature genes and molecular mechanisms in keratoconus
Source: J Cell Mol Med. 2024 Sep 19;28(18):e70079. doi: 10.1111/jcmm.70079 (PMC11412914; doi:10.1111/jcmm.70079)
Supplement: Supplementary file 2 — Material S2. [file JCMM-28-e70079-s002.zip › 7.GSEA analysis/1.ARL11/KEGG_STEROID_BIOSYNTHESIS.html]

Details for gene set KEGG\_STEROID\_BIOSYNTHESIS[GSEA]

|  || Dataset | ARL11.group.cls#h\_versus\_l |
| Phenotype | group.cls#h\_versus\_l |
| Upregulated in class | h |
| GeneSet | KEGG\_STEROID\_BIOSYNTHESIS |
| Enrichment Score (ES) | 0.30068737 |
| Normalized Enrichment Score (NES) | 0.9131416 |
| Nominal p-value | 0.5889328 |
| FDR q-value | 1.0 |
| FWER p-Value | 1.0 |
Table: GSEA Results Summary

  

Fig 1: Enrichment plot: KEGG\_STEROID\_BIOSYNTHESIS      
 Profile of the Running ES Score & Positions of GeneSet Members on the Rank Ordered List

  

| SYMBOL | TITLE | RANK IN GENE LIST | RANK METRIC SCORE | RUNNING ES | CORE ENRICHMENT || 1 | CEL | na | 2807 | 0.250 | 0.1317 | Yes |
| 2 | LSS | na | 5251 | 0.159 | 0.1965 | Yes |
| 3 | SOAT2 | na | 5826 | 0.142 | 0.3007 | Yes |
| 4 | DHCR24 | na | 10961 | 0.048 | 0.1936 | No |
| 5 | DHCR7 | na | 11614 | 0.040 | 0.2090 | No |
| 6 | TM7SF2 | na | 11866 | 0.038 | 0.2338 | No |
| 7 | SC5D | na | 12707 | 0.030 | 0.2348 | No |
| 8 | EBP | na | 15832 | 0.005 | 0.1490 | No |
| 9 | NSDHL | na | 16375 | 0.001 | 0.1343 | No |
| 10 | CYP51A1 | na | 18218 | -0.012 | 0.0913 | No |
| 11 | SQLE | na | 18507 | -0.014 | 0.0947 | No |
| 12 | LIPA | na | 19086 | -0.017 | 0.0929 | No |
| 13 | HSD17B7 | na | 19955 | -0.023 | 0.0879 | No |
| 14 | FDFT1 | na | 22431 | -0.044 | 0.0543 | No |
| 15 | SOAT1 | na | 23712 | -0.059 | 0.0673 | No |
| 16 | CYP27B1 | na | 27024 | -0.109 | 0.0646 | No |
| 17 | MSMO1 | na | 29908 | -0.185 | 0.1389 | No |
Table: GSEA details [plain text format]

  

Fig 2: KEGG\_STEROID\_BIOSYNTHESIS      
 Blue-Pink O' Gram in the Space of the Analyzed GeneSet

  

Fig 3: KEGG\_STEROID\_BIOSYNTHESIS: Random ES distribution      
 Gene set null distribution of ES for **KEGG\_STEROID\_BIOSYNTHESIS**
